# Beyond reflection: Diffractive and intra-active interventions in health knowledge

**DOI:** 10.1177/13634593261468036

**Published:** 2026-07-22

**Authors:** Adele Moore

**Affiliations:** 1 Liverpool John Moores University, UK

**Keywords:** gender and health, technology in healthcare, theory, research methodology

## Abstract

This article, for Health’s 30th anniversary special issue, engages with feminist theoretical frameworks which have been used to interrogate the ways reproductive health knowledges are made, shared, and contested. The paper begins by critically revisiting the history of the Women’s Health Movement, illustrating how representationalist epistemologies can fall short in disrupting the material-discursive apparatuses that uphold dominant biomedical logics. Following this, the article explores what a diffractive, new materialist methodology can offer to this field. Haraway’s ‘virtual speculum’ and Barad’s ‘agential realism’ are explored as provocations for reimagining how we can engage diffractively with the mediation of reproductive bodies and technologies. This paper therefore argues that a diffractive, agential realist approach not only decentres human agency, but foregrounds the entangled processes through which knowledge and health are enacted.

## Introduction

As *Health* marks 30 years of publishing critical, interdisciplinary work on health, illness and medicine, this anniversary invites critical engagement with the ways in which the field’s conceptual frameworks have shifted, and what methodological tools might guide future inquiry. Over the past three decades, feminist scholarship has played a pivotal role in shaping critical understandings of health, challenging biomedical authority, and foregrounding questions of embodiment, agency, and power (Clarke and Olesen, 1999; Creager et al., 2001; Marchessault et al., 2000; Mol, 2002; Pols, 2014; Potter, 2001). Yet contemporary developments – particularly the digital mediation of reproductive health – call for renewed engagement with these feminist legacies (Algera, 2026; Cunha-Oliveira et al., 2024; Lupton, 2019; Lupton and Maslen, 2019; Maslen and Lupton, 2018; Schneider-Kamp and Takhar, 2023). The proliferation of digital platforms, apps and data-driven technologies has transformed how bodies, reproduction, and care are monitored, represented, and understood, demanding that we revisit earlier feminist critiques of embodiment and biomedical authority.

This article revisits the epistemological interventions of the Women’s Health Movement (WHM), a feminist health activism movement of the 1960s, which aimed to craft a feminist vision of health. Prior to the WHM, women were not afforded access to medical knowledge about their bodies; this information was maintained and controlled by doctors and health professionals (Morgen, 2002). The WHM focussed on attaining autonomy by reconfiguring who was able to observe and represent women’s bodies. Drawing on critical engagements with the WHM by Haraway (1997a, 1997b), Murphy (2012), and Tuana (2006), this article explores how the WHM, whilst facilitating vast progression in women’s health literacy, remained constrained within a representationalist framing, failing to disrupt dominant biomedical frameworks and power dynamics. Haraway’s (1997b) ‘virtual speculum’ and Barad’s (2007) ‘agential realism’ are therefore explored as provocations to reimagine how we can engage diffractively with the mediation of reproductive bodies and technologies. WHM activists sought to reclaim knowledge of the body through practices such as self-examination and collective health education. This article offers a diffractive return to the WHM to examine what can be gained when our analysis transgresses representations and attends to the process of materialisation.

The WHM radically transformed who could produce knowledge about women’s bodies, contributing to vital improvements in the ways women’s health is known and practiced (Marieskind, 1975). However, as this article will demonstrate, its epistemological assumptions remained grounded in representationalist logics. In this sense, the WHM remained constrained by the assumption that the body exists as a stable object, awaiting more accurate feminist description. Under representationalism, knowledge is assumed as a straightforward, direct reflection of a pre-existing reality (Trundle et al., 2025). Therefore, to attain women’s bodily autonomy, the WHM worked on crafting a more authentic account of the body. STS scholars have urged movement away from representationalist tendencies, proposing that the production of knowledge is constitutive, rather than reflective of the realities studied (Barad, 2007; Haraway, 1997a; Law, 2004; Woolgar, 1988). From this perspective, realities are not passively awaiting discovery, but rather, emerge through the material-discursive practices deployed to engage with them. The term ‘material-discursive’ emphasises that matter and meaning are co-constitutive (Barad, 2007). In the context of the WHM, this encourages an understanding of health realities not as produced by biological processes alone, nor through solely language or discourse. Instead, taking a material-discursive approach alerts our attention to the ways these realities are enacted through the entanglement of matter and meaning.

This article draws on the works of feminist new materialists, Haraway (1997a, 1997b) and Barad (2007) to explore how feminist health scholarship can move beyond representationalist framing by attending to the material-discursive practices through which bodies, technologies and knowledges are enacted. The WHM challenged traditional medical authority and autonomy within women’s health; refashioning biomedical visualising techniques to reconfigure who is able to see and speak about the body (Murphy, 2012). However, the movement did not interrogate the ontological assumption that bodies are pre-existing entities, and therefore failed to examine the processes through which bodies are materialised and known. The WHM maintained a commitment to producing more accurate, feminist representations of the body. A diffractive approach, therefore, attends to the material-discursive process through which health realities are enacted, tracing the entanglement of bodies, technologies, discourses and practices (Barad, 2007). This article returns to the WHM as a site through which the limitations of representational feminist epistemologies can be examined.

The article proceeds in two stages. First, I revisit the WHM, providing a brief history of the movement, before unpacking one of their most prominent interventions, the vaginal self-examination. This foundational feminist self-help practice facilitates an articulation of the WHM’s simultaneous epistemological innovation and reliance on representationalist assumptions about the body. After exploring the vaginal-self exam, I unpack how a diffractive, intra-active (Barad, 2007; Haraway, 1997a, 1997b) approach to crafting health knowledges facilitates an attention to the process of materialisation; moving away from the representational pursuit to produce more authentic accounts of supposed pre-existing phenomena. Adopting a diffractive approach shifts the focus away from whether a representation of the body is accurate, and towards the practices through which bodies become knowable in the first place. It therefore enables analysis of how technologies, institutions, forms of expertise, and embodied experiences participate in the production of health realities.

## Revisiting the women’s health movement

Emerging during the 1960s in the United States, the WHM was a network of feminist activists, health collectives and community groups, working to reconfigure the ways in which women knew their bodies and accessed healthcare (Marieskind, 1975). Initially gaining momentum through collective activism focussed on the legalisation of abortion, women’s bodily autonomy and independence were at the forefront of the WHM (Norsigian, 2019). The Boston Women’s Health Book Collective^1^ was a key initiative within the WHM; their publication, *Our Bodies, Ourselves* (2011), became a foundational resource for feminist health education. Drawing from knowledge generated through feminist health workshops with women in Boston, the collective championed women’s autonomy by facilitating access to information about their bodies, health and sexualities. Early editions included sections on reproductive rights and masturbation, as well as contraception, pregnancy and post-partum depression (Boston Women’s Health Book Collective, 2011). *Our Bodies Ourselves*, embodied the WHM’s broader commitment to enabling women to produce and share knowledge about their own bodies, rejecting the traditional biomedical frameworks.

The aims of the WHM were vast yet centred around the ‘demand for improved health care for all women and an end to sexism in the health system’ (Marieskind, 1975: 219). A significant success of the WHM was the introduction of patient package inserts. In the late 1970s, the WHM drew attention towards the lack of information provided to patients taking the contraceptive pill, attending to the various problems and concerns expressed by women using it (Norsigian, 2019). Barbara Seaman was a key figure in this campaign; her book, *The Doctor’s Case Against the Pill* (1969), illuminated the potential health risks associated with oral contraceptives and criticised the medical establishment for failing to adequately inform women about their side effects. Through collective action, the U.S. Food and Drug Administration was successfully pressured into providing an information pamphlet with the contraceptive pill, which detailed the risks, ingredients, and side effects associated. This intervention afforded women crucial information about the contraceptive pill, marking a significant shift towards the recognition of their right to informed consent.

## Into our own hands?

The WHM was united in the aim to reclaim reproductive knowledge and women’s bodily autonomy. The movement provided women access to medical knowledge through publications such as *Our Bodies, Ourselves*, but also sought to critically re-examine the assumptions and practices of traditional medicine (Tuana, 2006). The WHM enacted crucial material and epistemological changes to the governance and production of medical knowledge. In this section, I unpack the way the WHM deployed the vaginal-self exam to reconfigure the medical gaze towards a feminist, embodied vision. Through their collective efforts, the WHM helped to secure greater transparency in medical practice and challenged the authority of male-dominated institutions over women’s bodies. Following this, I explore how the vaginal-self exam enables an articulation of the epistemological limitations of the WHM more broadly. The movement was successful in reconfiguring who was entitled to know and represent the body, yet their interrogations remained confined to representationalist tendencies. Representationalism assumes that knowledge functions by accurately describing or reflecting a pre-existing reality (Barad, 2007). As a result, without interrogating this epistemological underpinning, the WHM did not fully attend to the wider socio-political context from which it emerged, and at times, contributed to the very structures it aimed to dismantle.

The WHM reimagined women’s health through self-examination sessions and women’s wellness self-help groups (Tuana, 2006). Committed to collective consciousness-raising, the WHM not only taught women traditional health knowledges, but further encouraged them to ‘trust their cognitive authority’ (Tuana, 2006: 14). Learning about and from their bodies through self-examination, the WHM privileged embodied ways of knowing the body which had not previously been deemed legitimate. Technoscientific tools of traditional medicine were not rejected by the WHM, but instead, reappropriated to aid the production of alternative, embodied ways of knowing women’s health. The speculum – a medical instrument which had been exclusively used by practitioners to access vision of the cervix – was a tool central to the WHM’s feminist knowledge project. The earliest iteration of the device was a pewter spoon with a bent handle (Shende et al., 2024). The device was developed into the two-armed speculum used today, which uses a hinged mechanism to push the vaginal walls open, enabling vision for the operator (Shende et al., 2024). The speculum quickly became an emblem of the WHM, retrieved from the grasp of medical professionals and placed into the hands of women to access internal corporeal vision.

Group vaginal self-examination sessions emerged as a staple practice within the WHM, transforming the speculum ‘from a gynecological tool of control and suppression into an instrument of liberation’ (Tuana, 2006: 14). Prior to its feminist refashioning, deployment of the speculum was restricted to medical professionals, to open up their patients’ vaginal walls during examinations. Houck (2024) attends to the power dynamics at play within vaginal examinations of the era, illuminating the vulnerable position of the female patient, being examined by male physicians, keeping tight hold onto information about women’s bodies. The traditional deployment of the speculum exclusively afforded medical professionals the privilege of internal visualisation, helping to maintain the hierarchy of knowledge. The WHM’s reappropriation of the device therefore reconfigured who could gain access to vision of the cervix, encouraging women to study their own and other women’s anatomy. The group vaginal-self examination sessions marked both the WHM’s resistance towards medical authority, and its prioritisation of women’s embodied expertise (Murphy, 2012).

Tuana (2006: 14) describes the self-examinations instigated by the WHM as a ‘practice of epistemological resistance’; challenging dominant ideas about who can produce knowledge about the female body, where this takes place and which kinds of knowers are deemed valid within biomedical discourse. The vaginal-self examination exemplifies the core aims of the WHM: reclamation of bodily knowledge, reappropriation of technoscientific tools and enabling women to become active producers of knowledge about their own health. The WHM’s vaginal-self examination is thus a pertinent site for epistemological analysis, facilitating an effective articulation of the representationalist assumptions the WHM relied upon (Murphy, 2012). Women were encouraged to observe each other, to draw comparisons and similarities, getting to know female anatomy, one cervix at a time. The WHM emphasised a romanticisation of the vaginal self-examination, a collective process of demystification. Women were encouraged to ‘insert with care’ and ‘enjoy the lush color’, to admire their corporeality (Murphy, 2012: 68). The vaginal self-exam presented a defiance of the epistemological authority of medicine, as well as the kind of knowledge it produced about what constituted normal physiology.

Through the vaginal self-exam, the WHM challenged the traditional biomedical perspective by reconfiguring the gaze. This was no longer vision from the detached position of a doctor or medical visioning tool, but rather, the hands of the woman gazing inwards, becoming familiar with her anatomy. Traditional portrayals of the female body were disembodied; ‘arms, legs, and head severed’ (Murphy, 2012: 76). The self-examination facilitated a kind of vision which was situated and locatable, thus a discernible ‘learn[ing] in our bodies, endowed with primate color and stereoscopic vision’ (Haraway, 1988: 582). Women were encouraged to use their fingers, their senses, feelings and curiosity to lead their corporeal inquiry. The vaginal self-exam thus facilitated a deliberate, embodied departure from traditional modes of observation and detached vision, corresponding with feminist knowledge epistemological advances of the time. Murphy (2012: 98) observes, ‘relatives, if not the direct offspring’, of the vaginal self-exam can be found in the works of feminist standpoint scholars such as Smith (1974), Hartsock (1983), Harding (1991, 1992) and Collins (2014). Standpoint epistemology, a foundational feminist framework, challenged the myth of neutral, impartial knowledge developed from a distance, through proposing that knowledge is deeply shaped by an individual’s social location. Championing a ‘thinking from’ approach, the WHM and standpoint epistemology contested traditional notions of objective knowledge produced from afar, facilitating a reconfiguration of how knowledge is produced and who is able to participate in this process.

Whilst the WHM instigated a significant shift away from the patriarchal confines of traditional medical knowledge production, Murphy (2012) draws our attention to the ways that feminist self-help was intertwined with the historical circumstances of its development. The WHM destabilised existing power structures within medicine through co-opting biomedical technologies, yet Murphy (2012: 32) encourages an examination of the ways these practices were shaped by ‘contradictory conventions of stratifying and politicizing living-being’ such as race, citizenship and labour. In this sense, the movement cannot be understood as existing outside of the structures worked to contest, because its knowledge practices emerged through, and remained entangled within, the broader social, political and material arrangements. The WHM was therefore ‘both a *symptom and diagnosis* of its moment’ (Murphy, 2012: 32, emphasis in original). The WHM successfully identified the foundational problems associated with medicine; highlighting the ways women were excluded from the production of medical knowledge, as well as illuminating the paternalistic, sexist tendencies of medicine. The movement ultimately drew attention to the ways reproductive health was governed through unequal relations of expertise and authority. Yet, the practices of the WHM were not abstract nor detached, rather, they were enmeshed within the lived, material realities of the women who engaged with their practice. It is therefore crucial to acknowledge that the WHM emerged within a broader context of neoliberal restructuring, where responsibility for health was increasingly relocated from collective institutions to self-regulating subjects; shaping emerging understandings of autonomy, knowledge and bodily governance (Murphy, 2012). Whilst the WHM advocated for embodied knowing through consciousness-raising group sessions and self examinations, it remained within the limits of representationalist assumptions about knowledge production. This is because the WHM’s central political project was about asking who was able to know and represent the body, rather than unpacking the processes through which bodies, technologies and knowledges come to matter. From this perspective, the body appears as a pre-existing object awaiting more accurate or politically just forms of representation. Attending to the process of materialisation of bodies shifts the analytical focus from questions of representation to questions of emergence. Instead, asking how particular bodies come to matter through specific material-discursive practices, technologies, institutions and relations of power. Restricted to the project of producing more accurate, feminist representations of the body, the WHM neglected the broader political and economic conditions which shaped their knowledge practices, including the emergence of neoliberal forms of governance.

Murphy (2012) expands the analysis of the vaginal self-examination past romantic accounts of collaborative learning; attentive to the ways the feminist health vision was ‘formed through contradictions’, as practitioners ‘were simultaneously hailed as representatives of a politicized class (women) and as singular individuals’. This tension makes visible the limitations of understanding the WHM solely as a project of epistemic inclusion. Whilst the movement successfully challenged who could produce knowledge about women’s bodies, it paid less attention to the broader political and material conditions through which particular forms of embodiment, autonomy and health subjectivity were being constituted. Unpacking this tension raises a broader challenge for feminist health scholarship: how can we remain attentive to embodied experiences and material realities whilst also tracing the racialised, gendered, and classed relations through which those experiences are constituted? This contradiction urges feminist health scholars to stay with the trouble (Haraway, 2016), rather than striving for neat reconciliations and resolutions which quickly become obsolete. Therefore, though the vaginal self-exam was understood to have a ‘powerful consciousness-raising effect’, the scope of its success was restricted (Murphy, 2012). Favouring sexed, individualised embodiment, the WHM overlooked the more complex, intersecting, and complicit dynamics that shape subject formation in knowledge production. In promoting the pleasure of embodied inward gazing, the WHM established an epistemic privilege (Tuana, 2006). Yet the WHM failed to interrogate the unequal, material structures in place at the time, such as racism, poverty and ableism, instead encouraging a commitment to the similarities; knowing through drawing corporeal comparisons.

The WHM thus emphasised inherent femaleness as a core, collective, unifying factor (Murphy, 2012). The singularity of this category worked to erase and obscure the multiplicity of women’s identities, as crucial aspects of their lived experiences, such as race and class were pushed out of sight. The WHM did not transgress the indulgence of naval gazing to interrogate the interlocking systems of race, class, sexuality, ability and coloniality which shape and inform subjectivities. Resonating with early feminist standpoint epistemology interventions, the WHM began with the assertion ‘that all knowledge production should begin with women’s experiences’ (Murphy, 2012: 80). Phrases like ‘I saw this’, ‘I was there’, ‘I felt that’, declared at feminist self help meetings, worked to assert a ‘purported epistemic privilege gained from the immediacy of observing one’s self’ (Murphy, 2012: 80). The WHM thus falls into the same ‘tempting’ trap identified by Haraway (1988: 576), whereby ‘no insider’s perspective is privileged’, because all are deemed equally legitimate. Whilst this intervention was crucial in challenging the exclusion of women from medical knowledge production, it retains a representationalist understanding of knowledge. Experience was positioned as a privileged route to truth, grounded in the assumption that women could gain more accurate access to a pre-existing bodily reality through direct observation and embodied engagement.

Haraway (1997b: 41) parodies the, essentialising flaw of the WHM and the vaginal self-examination, illuminating the deeply political histories associated with the practice:

More than a little amnesiac about how colonial travel narratives work, we peered inside our vaginas toward the distant cervix and said something like, ‘Land ho! We have discovered ourselves and claim the new territory for women’.

Haraway likens the investigative ethos of the WHM to that of colonial explorers discovering ‘new’ territory. By framing the female body as unchartered land, discovered through their use of the speculum, the WHM ignores the medical, racial and colonial histories already deeply inscribed on the body. The speculum has violent, racist roots; developed by 19th century gynaecologist Marion Sims, the speculum was used to conduct experiments on enslaved Black women (Sowemimo, 2023). In recent times, the speculum has been used to carry out forced gynaecological exams in places such as prisons (Divito, 2014) and airports (Begum, 2017), demonstrating the persistence of the speculum’s association with coercion and the regulation of female sexuality. In the context of consensual cervical screening, scholars have drawn attention to the anxieties, fears and pain described by patients undergoing the pelvic examination (Arrivillaga et al., 2023; O’Laughlin et al., 2021). O’Laughlin et al. (2021) call for improvements in patient care and support when carrying out screenings, offering material recommendations in practice, such as sufficient lubrication and using the correct size speculum. Caricaturing the WHM, Haraway (1997b) draws our attention to the dangers of framing embodied knowing as a conquest, and the ways this can echo imperial logics. Representationalism operates on the assumption that knowledge is a direct depiction of a pre-existing object, such as the body, awaiting description; stabilising the dichotomy of the knower and the known. Though the WHM reconfigured who could partake in the knowing, Haraway’s satire demonstrates how representationalist knowing, even in the context of feminist knowledge projects, can reproduce colonial discovery logics, assuming the body as something pre-existing, rather than mutually produced through material-discursive entanglements. The WHM privileged embodied knowing and contributed to vast developments in women’s knowledges and experiences of health, whilst ultimately reproducing representationalist notions about the body and knowledge production.

The WHM worked to challenge the authority of traditional medical knowledge, yet projects like the vaginal self-exam rested on the assumption that women shared a universal bodily experience which could be revealed through feminist inquiry. In this sense, their feminist self-help practices challenged the biomedical authority of medicine, reconfiguring who was able to create new knowledge, yet the WHM retained a representationalist model of knowing. Representationalism assumes that knowledge functions by accurately describing or reflecting a pre-existing reality (Barad, 2007). From this perspective, feminist intervention becomes a matter of producing more truthful, or authentic representations of women’s bodies. The WHM therefore attempted to replace the authoritative, detached medical gaze with an embodied feminist vision. However, the underlying assumption remained unchallenged; the female body existed as a stable object awaiting better representation. As a result, the material-discursive practices through which bodies, technologies, and knowledges themselves are constituted, were overlooked. It is this representationalist framing and approach to knowledge which diffractive approaches – particularly those developed by Haraway (1997a, 1997b) and Barad (2007) – seek to move beyond.

Although the WHM facilitated feminist vision, reclaiming it from medical authority, the approach still framed the body as a consistent, coherent object, waiting to be revealed. The vaginal self exam thus enacted the cervix as pre-existing object awaiting more authentic, feminist representation. The vision of the WHM was locatable and situated, a vast improvement on the detached medical observer from above. However, Haraway’s (1997b) satire illuminates how the WHM approached the body as something which just needed more accurate, feminist, embodied representation; once the cervix can be seen, it can be accurately known. The WHM reconfigured who had the authority to observe, and therefore what could be new considered valid knowledge. However, their impact remains constrained to representationalist logics, and as such the belief that our embodied accounts are direct representations of a coherent, pre-existing reality. Having examined key feminist interventions in the epistemology of the WHM, highlighting the limitations of representationalist approaches to health and knowledge, the discussion now turns to diffraction; considering how this new materialist framework offers new ways of analysing the active, material-discursive production of health and the body.

## Diffracting health knowledges

The term ‘diffraction’ was first introduced by physician, Thomas Young, in 1801, to describe a phenomenon discovered through his ‘double slit experiment’ which observed the way sunlight shone through two slits within a thick piece of paper (Young, 1804). The experiment informs how we know light particles today, demonstrating that light acts as a wave. In recent years, this concept has found prominence within feminist methodologies of scholars in the humanities, social sciences and transdisciplinary technology studies (Udén, 2018). To continue, I will explore how this term has been adapted by Haraway (1987, 1997a, 1997b) and Barad (2007, 2003) to interrogate the limitations of reflection and representation.

Haraway (1987) refashioned diffraction as a visualising metaphor, counter to reflection, to challenge the way we think about knowledge, responsibility and relationality. Thus, diffraction is a ‘mapping of the interference, not of replication, reflection, or reproduction’ (Haraway, 1987: 300). Diffraction emphasises that knowledge and meaning are produced through relations of difference rather than similarities or mirroring. In taking a diffractive approach, we attend to the effects of difference; the ways in which boundaries, identities, and meanings are continually enacted and reconfigured through interaction. Diffraction thus presents a pertinent intervention in reflective models of knowing, which tend to presume the dichotomy of an observer and a stable object of study.

Whilst reflexivity has been productive in advancing feminist inquiry, its material impact is limited (Haraway, 1988). As endorsed by standpoint epistemologists (Collins, 2014; Harding, 1991, 1992; Hartsock, 1983; Smith, 1974), reflexivity helped to trouble the authority of neutral, universal knowledge claims. However, the notion of acknowledging one’s position, locating our gaze through various feminist epistemological tools, is not enough to challenge the dichotomy of realism and relativism. Reflection and reflexivity will not undo the representationalist logics which underpin and confine traditional knowledge practices. Haraway (1997a) calls for us to do more than reflect, positing that we need a reconfiguration of our tools, our research methods, which accounts for the ways our knowledge is produced. Thus, Haraway illuminates our methods as a performative apparatus, entangled within the world we are studying. Rather than seeking supposed mirror reflections of a stable reality, Haraway advocates for methods as apparatuses which enact and engage with boundaries. In this sense, research becomes about tracking and tracing difference and interference, asking what kinds of knowledges are enacted when different perspectives meet and converge. Resisting reflection, diffraction involves actively challenging the inward gazing which feminist inquiry has often relied upon and reverted to (Harding, 1991). Thus, Haraway’s (1988) diffractive intervention helps to reconcile feminist ‘efforts to climb the greased pole leading to a usable doctrine of objectivity’ (p. 580). Rather than attempting to climb this pole and hold on to both slippery ends, diffraction encourages a ‘mapping of the interference, not of replication, reflection, or reproduction’ (Haraway, 1987: 300). Thus, diffraction invites an analysis that attends to the overlapping forces, histories, and material conditions that shape the production of knowledge and subjectivity.

Reconfiguring feminist vision, Haraway (1997b) proffers the ‘virtual speculum’, a tool for doing diffraction. The virtual speculum attends to the situated, entangled nature of vision and the various technologies associated. In the context of the WHM, the lively vision of the luscious cervix, though seemingly a totally natural occurrence, exists ‘because of, and inside of, technoscientific visual culture’ (Haraway, 1997b: 23). The visualisations we attain are facilitated by the technological apparatus deployed. The diffractive, interrogatory virtual speculum troubles the way that visualising technologies, such as ultra-sound transducers and speculums are adopted without contextualisation, disregarding the social and political context within which it is materialised. Haraway (1997b: 22) calls for a broadening of our understandings of ‘new reproductive technologies’ which involves more than the technologies and procedures alone, but the wider socio-technical landscape surrounding reproduction. Thus, Haraway’s (1997b: 41) virtual speculum asks:

Is this what feminists mean by choice, agency, life and creativity? What is at stake here, and for whom? Who and what are the human and non-human centres of action? Whose story is this? Who cares?

The virtual speculum is a provocation for vision which contextualises modern visualising devices, exploring the wider network within which they exist and are known (Haraway, 1997b). The virtual speculum asks who is using them, why they are being used and what this is supposed to mean. This crafts a kind of vision which attends to the entanglement of agencies which enact our vision, and therefore our object of study. The virtual speculum records ‘interfering and shifted – diffracted – patterns’, as opposed to singular, one-way reflections, which might attempt to block out or silence interference (Haraway, 1997b: 41). The virtual speculum is not an approach in presenting a complete, absolute truth about the body, but rather, emphasises the value in knowing the kinds of patterns and interferences which occur when a range of differing perspectives, histories, agencies and materialities converge and clash. The virtual speculum aims for a work in progress, not stable resolutions.

Barad (2007) shares Haraway’s (1997b) disdain for reflexivity. Whilst the tradition of feminist inward gazing ‘has been recommended as a critical practice’, Barad (2007: 71) proposes that ‘reflexivity, like reflection, only displaces the same elsewhere, setting up worries about copy and original and the search for the authentic and really real. . .’. In this sense, reflexivity keeps us stuck in a continuous cycle, as we observe the same images being bounced back and forth. The aim, for Barad (2007: 88), is to disrupt the reliance on reflection, which is ‘set up to look for homologies and analogies between separate entities’. Barad urges for a tracing of interference and difference as a way to attend to how realities are materially enacted. Rather than searching for the most authentic reflection, we must attend to the dynamic, tentative materiality, tracing how phenomena come to matter through the entanglement of human and non-human actants. In this sense, reality, matter, phenomena, do not pre-exist our analysis, but are rather enacted through it.

Haraway’s (1987, 1997a, 1997b) deployment of diffraction operates within semiotic analysis; using diffraction as a tool for engaging with meaning-making, interrogating the politics of representation. Barad (2007: 416) transgresses this by attending to ‘the details of diffraction as a physical phenomenon and a methodology’. For Barad (2007), diffraction is about deep engagement with the material; advocating for a complete reconfiguration of discursive analysis to attend to the entanglements of matter and language, exploring the ways in which matter and meaning come to constitute one another. Barad’s deployment of diffraction offers an important intervention in the way we approach the study of health and medicine, challenging the detached, quantifiable biomedical approach and encouraging an attention towards the relational, situated, and entangled nature of knowledge-making practices. For Barad (2007: 91, emphasis in original), ‘*practices of knowing are specific material engagements that participate in (re)configuring the world’*. It is not enough for knowledge production to be about simply ‘making facts’ rather, we must explore the practice of ‘making specific worldly configurations’ (Barad, 2007: 91). Barad therefore illuminates the tentative, performative nature of fact, emphasising the importance of approaching knowledge making as a process of enacting realities, rather than reflecting pre-existing, stable phenomena.

Having outlined Haraway’s (1987, 1997a, 1997b) and Barad’s (2003, 2007) conceptualisations of diffraction as a methodological intervention, the discussion now turns to Barad’s agential realist framework. This provides a way of thinking beyond representationalism by attending to the ways matter, discourse, and knowledge emerge through entangled relations, opening up new possibilities for the study of health.

## Agential realism

Agential realism is Barad’s (2007) philosophical framework which proposes that reality is not comprised of pre-existing objects or subjects awaiting representation. Rather, agential realism suggests reality emerges through a series of ‘intra-actions’. Counter to the more conventional ‘interactions’, Barad (2007) proposed ‘intra-actions’ – dynamic relations between human and non-human agencies. Intra-actions promote an attention towards the active role of the observer in the constitution of reality. We cannot simply sit back and observe phenomena; rather, we must acknowledge the role we play in accounting for and engaging with the realities produced, we must meet our object of inquiry halfway. Barad thus articulates agency as a continuous process of becoming, involving human and non-human actants.

Discursive accounts of society and the body have been effective in illuminating the ways that medicine constructs bodies (Butler, 2006; Grosz, 1994; Martin, 1991, 1992). However, agential realism alerts us to what is lost when our analysis is limited to language; as matter is understood as ‘passive product of discursive practices’ (Barad, 2007: 151). In the context of the WHM’s interventions, the body is posited as a blank slate, awaiting cultural and social inscriptions. For Barad (2007: 151), this passive theorisation of matter discounts the ‘causal factors of materialization’ and offers a limited understanding of the dynamic processes associated with discursive practices. This bounds feminist engagement with medicine and science to the confines of criticism, and in turn, restricts understandings of discourse itself. Barad (2007: 151) therefore urges a reconsideration of matter, from ‘product of discursive practices’ to ‘an active agent participating in the very process of materialization’. This approach radically disrupts the binary between language and materiality that underpins much of Western epistemology. By attending to the agency of matter, Barad aligns with feminist new materialist and semiotic critiques that seek to move beyond representation (Alaimo and Hekman, 2008), emphasising instead the generative entanglements of discourse, embodiment and technology. From this perspective, the speculum is an active participant in the materialisation the body. Its materiality contributes to the production of meanings about the femininity, technology and visibility, illustrating Barad’s (2007) claim that matter itself participates in the making of the world. The cervix, when revealed through the WHM’s deployment of the speculum, is not uncovered; rather, it is enacted through the intra-action of bodily tissues, medical technologies, clinical practices and historical relations that make certain bodily realities visible and meaningful.

An agential realist reading of the WHM encourages a shift away from the question of whether the feminist self-help movement produced more authentic, accurate representations of women’s bodies, towards the material-discursive arrangements through which those bodies became knowable. Engaging with the vaginal self-examination practices of the WHM through Barad’s (2007) framework illuminates the limitations of the representationalist approach deployed by the movement. Rather than approaching the cervix as a pre-existing entity that becomes visible through feminist observation, agential realism encourages us to ask what makes the cervix emerge as an object of knowledge? The speculum, the mirror, the collective setting of feminist self-help groups, feminist health discourse and broader sociopolitical structures intra-act to enact the cervix as a visible, anatomical object. In this sense, intra-action emphasises that the cervix is not pre-existing, awaiting feminist discovery, but instead, materially enacted though these various actants. This approach shifts the analysis away from representation, which asks who is allowed to observe and describe the cervix, towards the material-discursive entanglements which reproductive bodies become knowable in the first place. The cervix becomes a phenomenon produced through tentative, specific intra-actions of matter and discourse rather than a stable object awaiting observation.

The WHM emphasised the importance of producing more authentic, feminist accounts of women’s bodies, whereas an intra-active approach encourages us to explore the material-discursive arrangements through which those bodies became knowable in the first place. Intra-action tells us that entities do not pre-exist their relations, but instead, emerge through them (Barad, 2007). In the context of the vaginal self-exam, a woman observing her cervix is not an independent subject observing an independent object. Rather, the subject and object are co-constituted; the feminist knower, the cervix as a visible object, and the authority of experiential knowledge emerge together. This approach encourages an examination of the tentative, material-discursive entanglements which constitute phenomena, moving away from representations, towards the process of materialisation.

The WHM worked to reclaim women’s knowledge of the body through deploying feminist visualising practices, rejecting the traditional medical accounts of knowing and doing women’s health. By providing women with the tools and space to observe and interpret their own reproductive anatomy, the WHM facilitated a rejection of the paternalistic practices of traditional medicine, enacting the located, embodied feminist gaze. This practice reconfigured who had the authority to create knowledge about women’s health. However, this intra-active reading of the vaginal self-examination illuminates the way that the WHM remained situated within to representationalist notions about the production of knowledge.

## Conclusion

In marking Health’s 30th anniversary, this article has revisited feminist theoretical engagements with reproductive health knowledges. By tracing the limits of representationalist epistemologies within the Women’s Health Movement, I have shown how approaches that seek to ‘speak for’ or ‘reveal’ can inadvertently reproduce the very material-discursive structures they aim to disrupt. Turning instead to a diffractive, new materialist methodology has allowed for a more nuanced account of how reproductive bodies, technologies, and knowledges are co-constituted. This article does not diminish the political achievements of the WHM, rather, illuminate the epistemological assumptions which shaped its interventions. Whilst the WHM reconfigured who could observe, and produce knowledge about women’s bodies, it remained tied to representational models of knowledge. In this sense, the goal remained focussed on creating more authentic, feminist accounts of the body.

An agential realist reading of the WHM has shifted the focus of our attention away from the task of producing more accurate representations, and towards an interrogation of the process of materialisation. Rather than asking who gets to observe, agential realism encourages us to explore the specific, tentative material-discursive entanglements through which bodies, technology and discourse emerge. Ultimately, this article argues that a diffractive, intra-active methodology offers a way of rethinking feminist epistemologies of health, encouraging an approach which is attentive to the ongoing, relational enactments through which knowledge, technology, and bodies take form. Such an approach invites continued experimentation with methods that do not reflect the world, but actively participate in its making.

## Data Availability

Any other identifying information related to the authors and/or their institutions, funders, approval committees, etc., that might compromise anonymity.

## Notes

1. The Boston Women’s Health Book Collective would later become known as ‘Our Bodies, Ourselves’.
